# Geographical Distribution of *Leishmania* Species of Human Cutaneous Leishmaniasis in Fars Province, Southern Iran

**Published:** 2013

**Authors:** M Akhoundi, H Hajjaran, A Baghaei, M Mohebali

**Affiliations:** 1 ANSES, JE2533 - USC «Transmission vectorielle et épidémiosurveillance de maladies parasitaires (VECPAR)», Université de Reims Champagne-Ardenne, Faculté de Pharmacie, France; 2 Parasitology Department, Pasteur Institute of Iran, Tehran, Iran; 3 Dept. of Medical Parasitology and Mycology, School of Public Health, Tehran University of Medical Sciences, Tehran, Iran; 4 Center for Research of Endemic Parasites of Iran (CREPI), Tehran University of Medical Sciences, Tehran, Iran

**Keywords:** *Leishmania tropica*, *Leishmania major*, PCR-RFLP, Iran, Human

## Abstract

**Background:**

The goal of this study was to know the identity of *Leishmania* species responsible of cutaneous leishmaniasis (CL) in Fars Province, southern Iran.

**Methods:**

Five counties of Shiraz, Firouz Abad, Ghir-Karzin, Farashband and Larestan were prospected. Forty-four patients exhibiting cutaneous lesions were selected. Samples collected on skin lesions were examined both microscopically (after Giemsa staining) and molecularly (after PCR-RFLP).

**Results:**

On the 44 examined patients, 39 exhibit *Leishmania* sp. by microscopical examination, all confirmed by PCR. For five patients with negative microscopical examination, PCR was positive for three of them. Among these 42 positive samples, 3 (7%) were infected by *L. tropica* and 39 (93%) by *L. major*.

**Conclusions:**

*Leishmania major* is the most prevalent species in prospected area and *L. tropica* occurs in Shiraz and Ghir-Karzin counties.

## Introduction

Leishmaniases are one of ten most important tropical diseases in the World. They occur as cutaneous, visceral and muco-cutaneous clinical forms. These diseases are recorded in Asia, Europe, Africa and America (1–3). In Iran, both visceral leishmaniasis due to *Leishmania infantum* and cutaneous leishmaniases constitute important health problems. Cutaneous leishmaniasis (ZCL) due to *L. major* (Kinetoplastida: Trypanosomatae) is a zoonotic disease with rodents as reservoirs. It occurs in rural regions of 15 out of 31 provinces of Iran (4). Most important endemic foci of this disease are located in Turkmen Sahara and Lotf Abad in north-east of Iran, Abardezh Varamin, Esfahan and Yazd in center of Iran, Fars and Sistan-Baluchestan in south and south-east, Ilam and Khuzestan in south-west of Iran (5–9). Cutaneous leishmaniasis (ACL) due to *L. tropica* is mainly an anthroponotic disease which occurs in Khorasan-e-Razavi in north-east, Tehran in center, Fars and Kerman provinces in the south of Iran (5, 7).

Fars province is an important focus of leishmaniasis (accompanying with Kerman Province) in south of Iran where *L. infantum*, *L. major* and *L. tropica* are circulating (5, 7, 10–12). According to the report of Iranian Ministry of Health, 23% (N = 5280) of annually reported cases of CL in Iran in 2009 were recorded from Fars Province (13).

Several studies have been carried out in Fars Province focusing mainly on the suspected patients of CL in Shiraz, Arsanjan and Jahrom Counties and also on the lesions of cutaneous patients of Fars Province (and probably its suburbs) that refer to health centers of Shiraz without naming the regions that patients come (14–18).

Surprisingly, considering to importance of Fars Province as a focus of cutaneous and visceral leishmaniases, little is known about the identity of *Leishmania* species responsible of human cutaneous diseases particularly in center and south of this province.

However, to our knowledge, there is a lack of data (human, reservoirs and vectors) in the counties of Firouz Abad, Ghir-Karzin, Farashband and Larestan. The goal of this study was to know the identity of *Leishmania* species responsible of CL in the counties of Firouz abad, Ghir-Karzin, Farashband and Larestan in comparison with the county of Shiraz.

## Materials and Methods

**Fig. 1:**
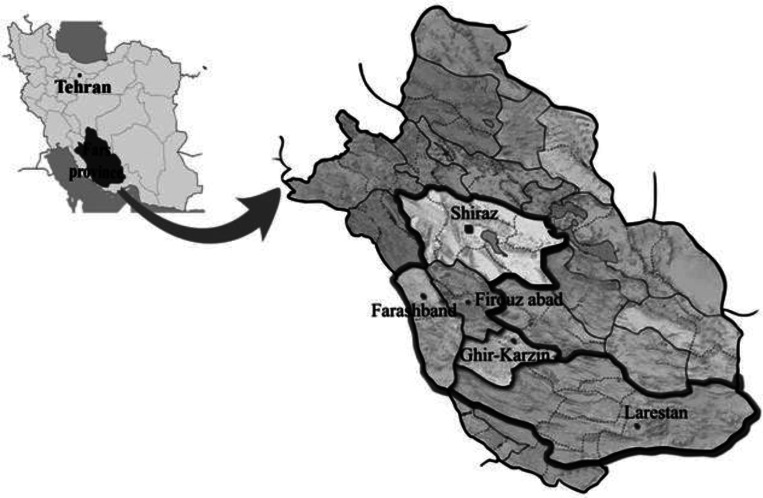
Sampled regions for suspected patients of cutaneous leishmaniasis in Fars province in southern Iran

Fars Province is located in south of Iran (27° 2’ N to 31° 42’ N and 50° 42’ E to 55° 36'E). A prospective study carried out since 18^th^ to 27^th^ October 2011 recording the cases of CL in 25 villages located in five Counties. They include: Shiraz (Gachi, Soltan Abad, Lapo-Oyeh, Shurijeh & Ghalat), Firouz Abad (Parzeitun, Ahmad Abad, Jaydasht, Khajei & Sarger), Ghir-Karzin (Ghir, Band-e-Bast, Marand, Eslam Abad & Mobarak Abad), Farashband (Dezhgah, Nojin, Bachun, Aviz & Mansour Abad) and Larestan (Zarvan, Fadagh, Karishki, Mahalche & Baladeh) counties (Fig. 1).

Patients were selected according to the data of the health center of each village who indicate persons exhibiting cutaneous lesions.

Forty four patients have been selected: Shiraz (11 patients), Firouz abad (6 patients), Ghir-Karzin (5 patients), Farashband (4 patients) and Larestan (18 patients). All of them exhibited one lesion whereas three exhibited two lesions. A total of 47 smears were prepared from active skin lesions of these patients. The personal information, lesion duration, type of lesion, lesion location, travels of patients to endemic regions and also drug consuming were recorded for each patient.

Cutaneous samples were prepared based on Evans protocol (19) and the samples were then smeared on a microscopic slide, air dried, fixed with absolute methanol, stained by Giemsa10% and examined under a light microscope with high magnification (1000X). The positive smears were scored for amastigote frequency according to the World Health Organization recommendations (20).

All smears were extracted using phenol-chloroform protocol followed by ethanol precipitation (21). The DNA was resuspended in 30 µl TE 1X and stored at -20°C.

We selected a molecular marker able to detect the DNA of *L. major*, *L. tropica* or *L. infantum*. Amplification of 18s (partial sequence), first Internal Transcribed Spacer (complete sequence) and 5.8S rDNA (partial sequence) of the ribosomal DNA has been carried out by PCR using the primers LITSR (forward: 5’-CTGGATCATTTTCCGATG-3’) and L5.8s (reverse: 5’-TGATACCACTTATCGCACTT-3’) under conditions described by Schönian et al. (2003) (22, 23). The length of amplicons ranked to 300-350 bp depending of *Leishmania* species. Negative and positive controls were used for each batch of PCR. Amplicons were analyzed using electrophoresis in 1.5% agarose gel containing ethidium bromide.

**Fig. 2:**
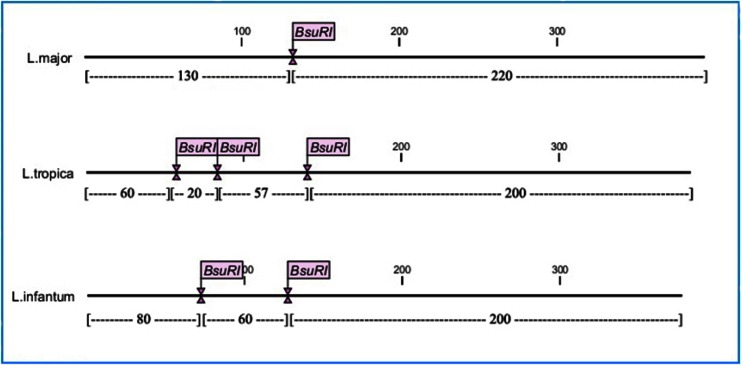
Cut sites of *Bsu*R1 (*Hae*III) in amplified fragments of ITS-rDNA in different *leishmania* species (CLC DNA Workbench 5.2 software)

The best enzyme for RFLP diagnosis has been chosen using CLC DNA Workbench 5.2 software (CLC bio A/S, Aarhus, Denmark). *Bsu*RI (=*Hae*III) was selected with cut site GG↓CC and cut *Leishmania* DNA one, two or three times, depending of species. Standard strains of *L. major*, *L. tropica* and *L. infantum* were used as controls (Fig. 2).

Endonuclease digestion was performed in a volume 30µl include PCR product 10µl, *Bsu*RI enzyme (Fermentas) 2µl, 10x buffer 2µl and distilled water 16µl for 4 hours at 37°C. Two negative controls were used that one of them was without restriction enzyme and other one had no PCR product. The fragments were analyzed using electrophoresis on agarose gel 3% containing ethidium bromide versus DNA ladder 50bp (Fermentas).

The fragments of 220 and 130 bp for *L. major*, and the fragments of 200, 80 and 60 bp for *L. infantum* were observed. About *Leishmania tropica*, the expected fragments length according to the software analysis were 200, 60, 57 and 20 bp. Due to small size of fragment 20 bp and overlapping of two fragments with near molecular weight 57 and 60 bp, the observed fragments length on the gel were 200 and 60 bp. Therefore, according to difference in number of cut sites (*L. major*:1, *L. tropica*:3 and *L. infantum*:2), number of fragments (*L. major*: 2, *L. tropica*: 4 and *L. infantum*: 3) and the fragments length (*L. major*: 220 and 130 bp; *L. infantum*: 200, 80 and 60 bp and *L.tropica*: 200 and 60 bp), *Leishmania* species were identified (Fig. 2).

Moreover, in order to confirm the results, two PCR products were directly sequenced both directions using the L5.8s and LITSR primers.

## Results

According to microscopical examination, 42 smears were positive for *Leishmania* sp. They belong to 39 patients (three patients exhibiting two lesions). Concerning five patients, no *Leishmania* have been observed (Table 1).

**Table 1:** Studied suspected patients of cutaneous leishmaniasis in Fars province

Grade	Number of smears	Patient sex		Lesion location				Cases with more than one lesion
		Male	Female	Hand	Foot	Head	Body	
(0) per 1000 field 0	5	2	3	2	3			
(1-10) per 1000 field +1	9	5	4	3	5		1	
(1-10) per 100 field +2	7	4	2	1	4	1*	1	foot (1) - foot (1)
(1-10) per 10 field +3	12	7	5	5	4	1*	2	
(1-10) per field +4	5	2	2	2	3			hand (1) - foot (1)
(10-100) per field +5	6	3	2	2	2	1	1*	hand (1) - foot (1)
(>100) per field +6	3	1	2	1	1		1	
Total	47	24 (54.54%)	20 (45.46%)	16 (34%)	22 (46.8%)	3 (6.4%)	6 (12.8%)	

* include infected sample with *L. tropica*

The processing of these samples by molecular techniques showed that all the 42 samples for which *Leishmania* had been observed were also positive by PCR. Concerning the five lesions considered as negative after microscopical examination, three extracted smears were positive by PCR. Consequently, the sensitivity of microscopical examination versus PCR is 93.3% in the present study.

On these 45 PCR positive smears, 42 were identified as *L. major* whereas only three were identified as *L. tropica*. The later come from Shiraz (N = 2) and from Ghir-Karzin (N = 1).

About the three patients exhibiting two lesions, all of them were positive and typed as *L. major*.

Most lesions due to *L. major* were located on the feet whereas the lesions due to *L. tropica* were located on the head (forehead and ear) and body (back). Most studied smears were 3+ and 1+ in amastigot grading index (Table 1).

Studied patients had an age range between 4 to 46 years old and most abundant lesions in this study were related to patients with 9-19 years old (61%).

The identification of two samples has been confirmed by direct sequencing of 10 µl of the PCR product. A BLAST comparison (www.ncbi.nlm.nih.gov/BLAST) showed 100% homology with standard strains of *L. major* and *L. tropica*, respectively.

## Discussion

In the past, detection of *Leishmania* sp. was based on morphological characters of parasite. Also, specific identification was not satisfactory. It depended on several factors such as the geographical distribution of an isolate, the clinical finding of the disease and the epidemiology of the vector and the animal reservoir (24, 25) with many problems in foci where two or more species were found in sympatry. The use of molecular methods since the 1990's, easier to use than the isoenzymatic typing in routine, changed the diagnosis providing a fast, sensitive and specific method. Ribosomal and kinetoplast DNA markers are commonly used. Schönian et al. 2003 (22), Volpini et al. 2004 (26), Al-Jawabreh et al. 2004 (27) and Gadisa et al. 2007 (28) coupled different markers with RFLP for detecting and identifying the *Leishmania* species among stained smear or biopsy samples.

The most practical commonly used method for laboratory diagnosis of CL, is preparing Giemsa-stained smears from patient's lesion and microscopic observation of Amastigotes.

The use of stained smears from active lesions of patients having a CL has some advantages. The *Leishmania* detection is fast, practical and cheap but it needs trained slide readers. The transfer of the samples to the laboratory is easy to do. Very interestingly, the processing of these samples with molecular methods is possible: i) to identify the parasite at the species level, ii) to exclude any leishmaniasis for smears negatively examined by microcopy.

CL is characterized by one or more cutaneous lesions on areas where sandflies have fed. The morphology of the cutaneous lesions varies and may change in size and appearance over time depending of the *Leishmania* species, immune status, nutritional status, species and strain (29). The majority of patients (93%) processed in present study, had one cutaneous lesion predominately on exposed sites - varying in size from 0.5 to 2 cm in diameter - except three patients that carried two lesions contemporary.

Based on our findings, the majority (93%) of studied cutaneous patients in Shiraz, Firouz abad, Ghir-Karzin, Farashband and Larestan Counties were infected by *L. major* as well as it reported in some investigations which carried out in Shiraz, Arsanjan and Jahrom Counties in Fars Province (12, 14, 15).

All of prospected patients in present study were affected in their life places and had no travel to the other endemic foci of CL in Fars province as well as Iran.

In present study, most frequent lesions due to *L. major* were observed on the legs (47%) and then on the hands of patients (34%) whereas most common location of the skin lesions were reported on the faces and hands (Faces of school children (37%) & hands of all age groups (45.3%)) of patients in Arsanjan County (14). Also, three cutaneous patients infected by *L. tropica* were identified in the present study. Two of them were from Shiraz County.

It is interesting to note that a man infected by *L. tropica* comes from the county of Ghir-Karzin where no case has been previously recorded. The lesions of patients infected by *L. tropica* were located in head (forehead and ear) and body (back) parts.

According to our findings, the age range of patients with high prevalence of CL belonged to patients with 9-19 years old (61%) while in investigation which carried out among cutaneous patients of Arsanjan County (14), the most prevalence was related to patients above 15 years old (45%). Unlikely, in contrary with mentioned results, most prevalence of cutaneous lesions among patients examined in Shiraz (14.2%) and Jahrom (35%) Counties, belonged to children aged <10 years old (12, 15).

## Conclusion

Isolation of *L. major* as a prevalent species from suspected patients of CL in all prospected counties in present study, confirms these regions as ZCL foci as well as *L. tropica* -with lesser frequency- which was isolated from patients in Shiraz and Ghir-Karzin Counties as ACL foci in Fars province in south of Iran.

Cconsidering to importance of Fars Province as the one of most important foci of CL in Iran and considering to our findings, little is known about the disease reservoirs and sandfly vectors of *Leishmania* species, responsible of CL in prospected Counties in present study. Therefore, to carry out more parasitological and entomo-epidemiological investigations will develop our knowledge about CL circulation in mentioned regions.

Also, PCR-RFLP based genotyping assay is cheap, reliable with high sensitivity and specificity, easy to perform, and enables differentiation of the clinically relevant *Leishmania* species within a reasonable timeframe. It can be used as a suitable method for direct diagnosis and characterization of *Leishmania* species including *L. major* and *L. tropica* isolated from Giemsa-stained slides (even archived slides) while it can be carried out in other endemic foci of CL in Iran.
